# In vitro evaluation of a self-emulsifying drug delivery system (SEDDS) for nasal administration of dimenhydrinate

**DOI:** 10.1007/s13346-019-00634-1

**Published:** 2019-03-14

**Authors:** Christina Leichner, Randi Angela Baus, Max Jelkmann, Melanie Plautz, Jan Barthelmes, Sarah Dünnhaupt, Andreas Bernkop-Schnürch

**Affiliations:** Center for Chemistry and Biomedicine, Department of Pharmaceutical Technology, Institute of Pharmacy, University of Innsbruck, Innrain 80/82, 6020 Innsbruck, Austria

**Keywords:** Self-emulsifying drug delivery systems, Nasal administration, Emulsion, Dimenhydrinate, Permeation

## Abstract

The objective of the study was the development and in vitro characterization of a self-emulsifying drug delivery system (SEDDS) for the nasal application of dimenhydrinate. Final composition of SEDDS was established based on drug solubility and stability studies. Dimenhydrinate was loaded into the SEDDS pre-concentrates to 7.5% (*m*/*v*). The droplet size of the final SEDDS formulations was in a range between 60 and 220 nm. Permeability, as well as tissue toxicity, of the formulations was investigated using bovine nasal mucosa. Enhancement in permeation up to 2.8-fold compared to pure dimenhydrinate was confirmed. Furthermore, toxicity studies did not reveal any serious tissue damages related to the SEDDS. Additionally, irritation potential of SEDDS was evaluated in ciliary beat frequency measurements. Incorporation of dimenhydrinate into SEDDS might therefore be considered as a promising approach within the field of nasal delivery of antiemetics by utilizing permeation enhancement strategy.

**Electronic supplementary material:**

The online version of this article (10.1007/s13346-019-00634-1) contains supplementary material, which is available to authorized users.

## Introduction

In recent years, the nasal route has been increasingly investigated for the systemic delivery of drugs. Distinctive properties like the large surface area of around 150 cm^2^, extensive vascularization, high permeability, and the avoidance of the hepatic first-pass metabolism make the nasal cavity attractive as an application site [1, 2]. Especially, small lipophilic drugs are getting well absorbed by the nasal cavity with pharmacokinetic profiles close to those obtained after intravenous injection achieving bioavailability of nearly 100% [3, 4]. Marketed formulations for the nasal administration are used in the therapy of migraine (sumatriptan (Glaxo Smith Kline) and zolmitriptan (Astra Zeneca)), or for the treatment of pain (butorphanol (Bristol-Myers Squibb)) [3, 5]. Besides, a broad spectrum of nasal products is currently in the stage of development, covering therapeutic fields like epilepsy, rheumatoid arthritis, cancer therapy, insulin-dependent diabetes, and the delivery of antiemetics.

For the treatment of nausea and vomiting, where the use of the oral route might be complicated or not feasible, nasal administration offers a suitable alternative as a rapid onset of action is provided combined with convenient dosing [6].

Dimenhydrinate belongs to the class of antiemetic drugs and is approved for the prevention and therapy of nausea and vomiting [7, 8]. By reason of the fact that only small volumes can be administered via the nose, drug solubility plays an essential role within formulation design. As dimenhydrinate is poorly soluble in water, it was the aim of the present study to create a drug delivery system able to solubilize a sufficiently high payload for therapeutic needs. A promising approach facing this issue might be its incorporation into a self-emulsifying drug delivery system (SEDDS). These kind of carrier systems proved already their usefulness addressing other mucosal surfaces [9–13].

As far as we know, SEDDS have not been investigated within this scope yet, and therefore, it was the aim of the present study to create SEDDS for the nasal administration of dimenhydrinate followed by their characterization with regard to drug release, permeability across excised bovine nasal mucosa, and evaluation of the formulations´ tissue toxicity as well as irritation potential.

## Materials and methods

### Materials

Labrasol (caprylocaproyl polyoxyl-8 glycerides HLB 12) and Transcutol HP (diethylene glycol monoethyl ether HLB 4) were a gift from Gattefossé (France). Capmul MCM (caprylic/capric mono- and diglyceride HLB 5.5) and Capmul PG-8 (propylene glycol monocaprylate HLB 6.7) were a gift from Abitec (USA). Polyethylene glycol (PEG) 200 and 400 were obtained from Merck (Germany). Acetonitrile HiPerSolv Chromanorm and water HiPerSolv Chromanorm were supplied by VWR (Austria). Dimenhydrinate, Cremophor EL (polyoxyl 35 hydrogenated castor oil HLB 12–14), propylene glycol (PG), and all other substances and chemicals were purchased from Sigma-Aldrich (Vienna, Austria).

Dulbecco’s phosphate buffered saline (PBS): 2.67 mM potassium chloride, 1.47 mM potassium phosphate monobasic, 136.9 mM sodium chloride, 8.1 mM sodium phosphate dibasic; pH range 7.1–7.5; osmolality: 315 mOsm/kg.

Ringer’s solution: 148.3 mM sodium chloride, 4 mM potassium chloride, 2.2 mM calcium chloride dihydrate, 20 mM 4-(2-hydroxyethyl) piperazine-1-ethanesulfonic acid (HEPES) pH 7.4; osmolality: 342 mOsm/kg.

### Methods

#### HPLC method development for dimenhydrinate

Within this research, a suitable HPLC-DAD method for analyzing dimenhydrinate in pure samples, as well as in SEDDS formulations, was developed by considering all optimization parameters with a simple sample preparation. The HPLC system was a Hitachi EliteLaChrom HPLC with software EZ CHROME ELITE, L-2450 DAD detector, an L-2200 autosampler, and L-2130 pump, which ensured consistent system-to-system performance and high reproducibility. Separation was performed on a Multo-High Bio 200, C18 5 μm (250 × 4.6 mm) column including the same pre column at 35 °C. Mobile phase consisted of 20 mM ammonium acetate buffer pH 7.5, and acetonitrile (ACN) mixed in a volume ratio of 30:70. The flow rate of mobile phase was maintained at 1 mL/min for 8 min. Injection volume of each sample was set to 10 μL. The DAD detector was set at 225 as well as 273 nm. As standard stock solution, 1000 μg/mL of dimenhydrinate was prepared in a mixture of ACN/water (1:1), stored at − 20 °C in a glass flask and brought to room temperature before use. Standard solutions in the range of 3.125–500 μg/mL were daily prepared and filtered with a Millipore filter (0.45 μm) before transfer to the auto sampler plate for analysis. In case of SEDDS formulations, samples were diluted with ACN/water (1:1) in a ratio of 1:200, shaken for 30 min, centrifuged, and filtered before analysis. Single excipients of the SEDDS did not show a significant absorbance at the chosen detection wavelengths, and therefore, any disturbing signals could be excluded. Calculations were performed using a calibration curve constructed from standard dimenhydrinate solutions. The calibration curves are provided in the supporting information associated to the article (Fig. S1 and S2; Table S1 and S2).

#### Development and characterization of SEDDS

##### Solubility studies

SEDDS were developed on the base of drug solubility evaluation in various oils, surfactants, co-surfactants, and solvents. Single components were pipetted in 2 mL reaction tubes with a Pos-D micropipette (Mettler-Toledo, Switzerland) and mixed at 40 °C and 1000 rpm with an excess amount of dimenhydrinate using a thermomixer (Thermomixer comfort, Eppendorf, Germany). The amount of dissolved drug was determined by HPLC (Fig. S3).

##### Composition of SEDDS pre-concentrates

Excipients in which dimenhydrinate exhibited the highest solubility were combined in different volume ratios in order to obtain the most suitable SEDDS pre-concentrates (Table 1). For this purpose, excipients were mixed at 40 °C and 1000 rpm until a homogeneous phase was obtained. Pre-concentrates were visually investigated regarding phase separation, precipitation, or turbidity over a 24-h storage time at room temperature. Maximum payload of dimenhydrinate within the pre-concentrates was determined by HPLC.

**Table 1:** Composition of SEDDS pre-concentrates

	Components [%]							
	Transcutol HP	Capmul MCM	Capmul PG-8	Labrasol	Cremophor EL	PEG 200	PEG 400	PG
F1	15	20	–	–	25	20	–	20
F2	15	–	15	–	30	–	20	20
F3	20	10	–	30	20	–	10	10

##### Construction of pseudo-ternary phase diagrams

Phase diagrams were constructed with the aim to define the area of emulsion formation. Accordingly, homogeneous mixtures of surfactant (hydrophilic components with HLB > 10) and oily phase (hydrophobic components with HLB < 10) were prepared in volume ratios between 9:1 and 1:9. Afterwards, water was added drop by drop to each mixture under continuous stirring at 25 °C. Phase behavior was assessed visually. Continuous phases with a visual appearance between slightly bluish, bluish white less clear, and bright white [14] were classified as emulsions. Diagrams picturing the area of emulsion were mapped using the software Triplot version 4.1.2. Phase diagrams were constructed on the one hand for the blank formulations and on the other hand for the drug loaded formulations containing 75 mg/mL of dimenhydrinate.

##### Stability studies of dimenhydrinate loaded formulations

Final SEDDS formulations (F1-F3) were prepared by emulsifying the pre-concentrates in Ringer’s solution in a volume ratio 1:1. For further characterization and within the following experiments, SEDDS formulations were used in this dilution. SEDDS formulations were first examined for their stability during 2 weeks storage at 25 °C. Hence, particle size and PDI were recorded at time point 0 h, 3 h, 24 h, and 2 weeks. In addition, steadiness of the formulations was verified in a thermodynamic stress test with consecutive 24 h temperature cycles. After storage at 25 °C, probes were kept at a temperature of − 20 °C followed by the last cycle at 4 °C. Mean droplet size and polydispersity index (PDI) of the formed emulsions were determined at 37 °C by photon correlation spectroscopy at an angle of 173 ° (non-invasive back-scatter (NIBS)) with a Zetasizer Nano ZSP with a laser wavelength of 633 nm (Malvern, USA). The instrument has a range between 0.3 nm and 10 μm and an absolute sensitivity of 300 kcps (toluene).

##### Transmission electron microscopy

Morphology of SEDDS was characterized by transmission electron microscopy (TEM) using a Zeiss Libra 120 Energy Filter Transmission Electron Microscope (Zeiss, Germany) equipped with a TRS 2k × 2k high-speed digital camera (Tröndle, Germany). Samples were prepared by transferring 5 μL of the emulsion diluted 1:100 in demineralized water to a 400 mesh Formvar-Carbon coated copper grid. Subsequently, samples were dried before examination. Images were recorded with an Image SP software (Tröndle, Germany) employing a voltage of 80 kV at a magnification of × 6300.

#### Assessment of spreadability

Spreadability measurements were performed at 25 °C using a texture analyzer (TA.XT*Plus*, Texture Analyser Stable Micro Systems, UK) equipped with a TTC Spreadability Rig (HDP/SR). Briefly, 4 mL of the test material was filled into the female perspex cone-shaped product holder and after that the male cone was lowered with a speed of 3 mm/s up to a gap width of 2 mm. Force-time diagrams were recorded, and subsequently, the material’s firmness determined and the work of spreading calculated.

#### Sprayability test

The sprayability of the pre-concentrates was evaluated utilizing a conventional nasal pump spray device. Pre-concentrates comprising a dimenhydrinate content of 75 mg/mL were sprayed and the discarded volume was collected in a plastic tube. Probes were analyzed by HPLC. Efficiency of sprayability was determined with respect to the detected amount of dimenhydrinate within the liberated dose.

#### Determination of log P and log D

Maximum solubility of dimenhydrinate in the SEDDS pre-concentrates, in octanol, and in the emulsifying media was determined. Consequently, an excess amount of drug was dispersed in each particular test solvent and samples were shaken at 1000 rpm for 12 h at room temperature (Thermomixer comfort, Eppendorf, Germany). The amount of dissolved drug, in the supernatant, was analyzed after centrifugation (13,400 rpm, 2 min; MiniSpin, Eppendorf, Germany). The partitioning coefficient (log D) of dimenhydrinate between SEDDS (lipophilic phase) and the emulsifying or rather the release medium was calculated according to eq. 1 [15]. Octanol/water partition coefficient (log P) of dimenhydrinate was determined at room temperature by shaking the saturated mixture of the two phases for 24 h at 1000 rpm and analysis of the drug content in each phase after phase separation.

1 $$ \log D=\frac{c\ \left(\mathrm{SEDDS}\right)}{c\ \left(r\mathrm{elease}\ \mathrm{medium}\right)} $$

#### Preparation of bovine nasal mucosa

Mucosa was prepared according to a protocol described previously [16]. Briefly, bovine nasal mucosae were obtained from a local abattoir immediately after slaughter of the animals. Mucosal tissue was carefully excised from the lateral cartilage using a sharp knife, and directly used for the permeation and toxicity experiments.

#### Permeation across bovine nasal mucosa

Permeation of SEDDS formulations through bovine nasal mucosal tissue was investigated using Ussing-type diffusion chambers (Hugo Sachs Elektronik– Harvard Apparatus GmbH, Germany), following a method previously described [17, 18]. Therefore, excised tissue was cut into pieces of about 1.5 cm^2^ and was mounted on the chambers with the mucosal site oriented towards the donor compartment. The accessible permeation area was 0.64 cm^2^. Mucosa was 20 min preincubated with pre-heated Ringer’s solution by reasons of equilibration. One milliliter of SEDDS diluted in Ringer’s solution was filled into the donor compartment and 1 mL of pure Ringer’s solution was added to the acceptor chamber. Dimenhydrinate in the same concentration in Ringer’s solution was used as a control. For reasons of comparability, a concentration of 5 mg/mL dimenhydrinate was used in the donor chamber, as the pure drug is soluble in Ringer’s solution at this concentration. Chambers were incubated at 37 °C under continuous oxygenation and samples of 100 μL were withdrawn every hour from the acceptor compartment over a period of 4 h. The cumulative amount of permeated dimenhydrinate was expressed as a percentage of the initial amount of dimenhydrinate present in the donor compartment. Values of the apparent permeability coefficient (*P*_*app*_ values) were calculated according to eq. 2

2 $$ \mathrm{Papp}=\left(\frac{dQ}{dt}\right)\ast \left(\frac{1}{A\ast c}\ \right) $$

where *dQ*/*dt* represents the flux of dimenhydrinate (μg/s), *A* stands for the permeation area of the chamber (cm^2^), and *c* represents the initial concentration of drug in the donor solution (μg/cm^3^).

#### Evaluation of tissue toxicity

##### Lactate dehydrogenase assay

The amount of lactate dehydrogenase (LDH) released from fresh bovine nasal tissue during the permeation experiment in the Ussing chambers was determined with the CytoTox-ONE™ Homogeneous Membrane Integrity Assay (Promega Corporation, USA) according to the manufacturer’s instructions with slight variations. Briefly, after 4 h permeation study, 100 μL of the medium in the donor compartment was transferred to a 96-well plate containing 100 μL of CytoTox-ONE™ Reagent. Following incubation of 10 min at 22 °C, 50 μL of the stop solution from the kit was added to each well. After shaking for 10 s, fluorescence was recorded at an excitation wavelength of 560 nm and an emission wavelength of 590 nm (TECAN Infinite M200, Austria GmbH). As 100% control causing maximum LDH release served a 1% (*m*/*v*) Triton X-100 solution having been incubated with the tissue for 4 h prior to the assay.

##### Resazurin assay

The effect of SEDDS on tissue viability was examined performing a resazurin assay [19, 20]. In brief, fresh bovine nasal tissue was cut into pieces of 1 cm^2^ and was incubated with the SEDDS formulations for 4 h at 37 °C in a 24-well plate. Two concentrations were used to figure out any concentration dependent effect. On the one hand, the tissue was put into 300 μL of PBS and its luminal surface was overlaid with 10 μL of a 1:2 SEDDS dilution to simulate more closely in vivo-like coverage of the tissue, and on the other hand, the tissue was treated with 500 μL of a 1:50 dilution in PBS buffer. PBS served as negative control and a 1% (*m*/*v*) Triton X-100 solution as positive control. Following incubation, the tissue was washed with 2 mL of PBS, and 1 mL of a 5% (*m*/*v*) resazurin solution was added and for another period of 2 h incubated. Fluorescence of the supernatant was measured at 540 nm excitation wavelength and 590 nm emission wavelength (TECAN Infinite M200, Austria GmbH).

##### Ciliary beat frequency measurement

Ciliated epithelial cells were removed by nasal brushing of porcine nasal epithelium from the middle turbinate of freshly slaughtered pigs. After that, the initial ciliary beating of the cells was recorded at 23 °C utilizing a microscope (Motic Type AE31, Motic GmbH, Germany) connected to a high-speed camera system (Motion Scope M1, Imaging Solutions GmbH, Germany) to identify cilia in motion. Afterwards, porcine nasal epithelium was overlaid with the SEDDS pre-concentrates (10 μL/ cm^2^) and incubated for 20 min representing the average contact time of applied formulations on the mucosa in vivo under consideration of mucosal transit [21]. Subsequently, epithelium was rinsed with Ringer’s solution and brushed. Obtained cells were analyzed under equal conditions as outlined above in order to determine the impact of formulations on ciliary beat frequency (CBF). All videos were recorded with 500 frames per second for four seconds. For the calculation of CBF from the recorded AVI video files, a MATLAB algorithm was used including the following parameters. A region of interest (ROI) was first defined for each file for the purpose of improving the efficiency of the algorithm while excluding non-beating video sections. Each pixel within the selected ROI was analyzed regarding its standard deviation over time (SDIV) and was excluded if it was lower than a calculated threshold, defined as a factor 2 above the most frequent SDIV of all pixels. The remaining pixels were averaged in 3 × 3 pixel regions and a fast Fourier transformation (FFT) analysis was carried out using the smoothed pixel information. Videos were recorded with 500 frames per second for four seconds, resulting in frequency steps of 1 Hz from 0 to 250 Hz after FFT analysis. Every pixel’s frequency was determined as the highest amplitude in the frequency range from 0 to 20 Hz. The final CBF for each sample was determined as the most prevalent frequency in the histogram of all calculated pixels [22].

#### Statistical data analysis

The software GraphPad Prism version 5.01 was used for the statistical data analysis. One-way ANOVA and Bonferroni *t* test were performed with *P* < 0.05 as the minimal level of significance.

## Results and discussion

### Development and characterization of the SEDDS

#### Solubility studies

The solubility of dimenhydrinate was examined within the first step of the development of SEDDS in order to find the final composition of the pre-concentrates. All used excipients were of pharmaceutically acceptable quality. Dimenhydrinate possessed the highest solubility in Transcutol HP (HLB 4) followed by Capmul MCM (caprylic/ capric mono- and diglyceride HLB 5.5) and Capmul PG-8 (propylene glycol monocaprylate HLB 6.7). Accordingly, these three components were included into the formulations to build the oily vehicle for the drug. Cremophor EL (HLB 12–14) and Labrasol (HLB 12) are well-known, non-ionic, biocompatible surfactants often used in the preparation of SEDDS. Due to their high HLB value > 10, formation of o/w emulsions is favored. In general, at least 30% (*v*/*v*) of the formulation should be constituted by surfactants to achieve a fast and stable emulsification of the hydrophobic content. Co-surfactants are mostly beneficial for the system as they support the formation of fine droplets and the stability of the emulsion can further be preserved, as these substances lead to a reduction of the interfacial tension. Besides, co-surfactants can enhance drug solubility within the formulation. Hence, PEG 200 and PEG 400, as well as PG, were involved in the formulation design. All three excipients are common in intranasal formulations since they are nontoxic and non-irritant to the nasal mucosa [23].

#### Composition of SEDDS pre-concentrates

Aforementioned excipients were combined in different volume ratios and investigated visually for stability. The most promising pre-concentrates (Table 1) showing no changes over at least 24 h storage at room temperature were chosen for emulsification. An overview of different pre-concentrate combinations from preliminary investigations is given in Table S3 in the supporting information. Maximum extent of drug loading within the pre-concentrates was 336 mg/mL for F1, and 301 mg/mL in case of F2 and F3 (Fig. S4). Due to extensive first-pass effect of dimenhydrinate after oral dosing, 23–46 mg can be considered as the available drug amount of a recommended single dose of 50–100 mg [24, 25]. Under the assumption of a comparatively higher nasal bioavailability due to the avoidance of this first-pass metabolism and an applied volume of 100 μL per nostril, a drug load of 75 mg/mL per pre-concentrate corresponding to a nasal dose of 30 mg after two applications should be sufficient [26].

#### Construction of pseudo-ternary phase diagrams

**Fig. 1:**
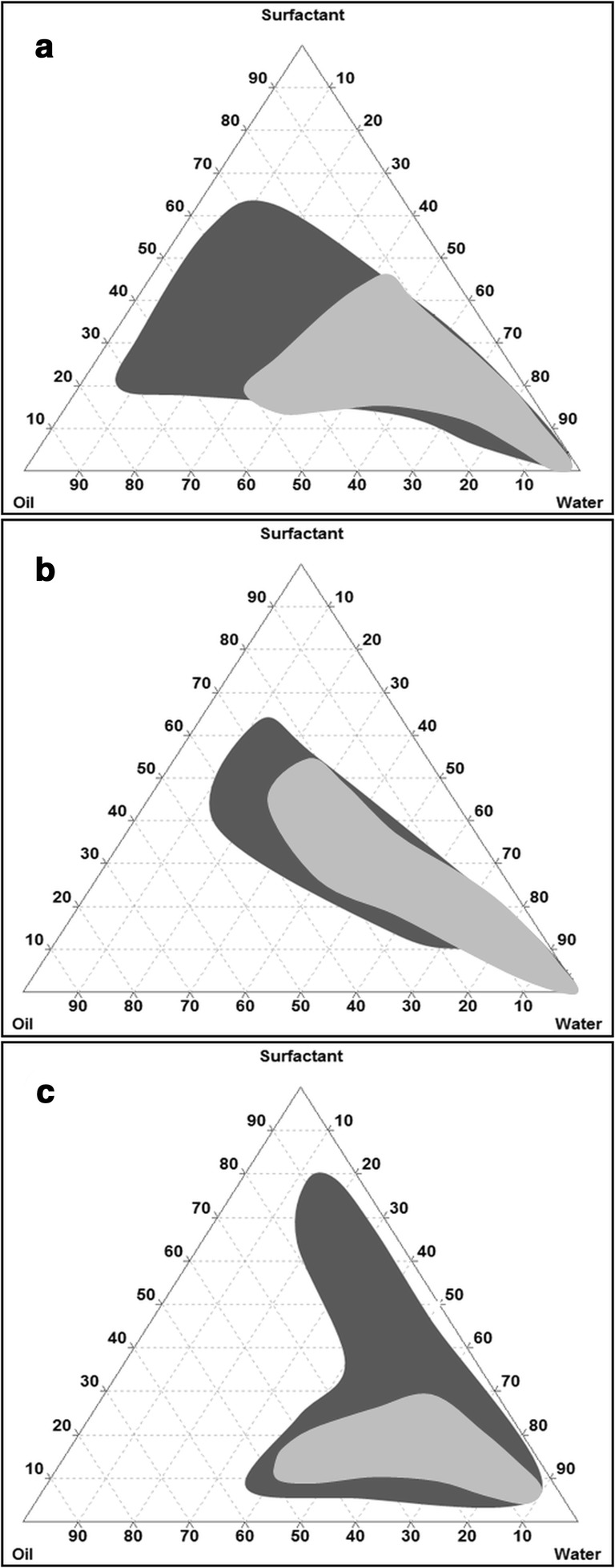
Pseudo-ternary phase diagrams of formulation F1 (**a**), F2 (**b**), and F3 (**c**). Components are represented in volume percent and the shaded areas depict the regions of emulsion. Diagram **a**: surfactant phase (Cremophor EL/PEG 200/ PG (1.25/1/1)), oil phase (Transcutol HP/Capmul MCM (1/1.3)); diagram **b**: surfactant phase (Cremophor EL/PEG 400/PG (1.5/1/1)), oil phase (Transcutol HP/Capmul PG-8 (1/1)); diagram **c**: surfactant phase (Cremophor EL/PEG 400/ PG (2/1/1)), oil phase (Transcutol HP/Capmul MCM (2/1)). Excipients were mixed at 25 °C. Dark gray area, blank formulation; light gray area, dimenhydrinate loaded formulation

Components are depicted in percent by volume. The gray areas represent the amount of surfactant, oil, and water necessary to form o/w emulsions (Fig. 1). Emulsions exhibited a visual appearance between slightly bluish, bluish white less clear, and bright white [14] and were formed in less than 2 min. Photographs picturing the visual appearance of emulsions according to this classification are presented in the supporting information (Fig. S5). The larger the emulsion area, the better are the emulsifying properties of the particular system. Pseudo-ternary diagrams of F1 (a) and F2 (b) were comparable except for the larger area of emulsion in case of F1. McConville et al. reported a similar behavior upon replacement of Capmul MCM by Capmul PG-8 [27]. Due to the combination of Labrasol and Cremophor EL in F3, the self-emulsifying area could be enlarged up to a higher lipid/surfactant ratio. These findings are in good agreement with the results of the particle sizes, as F1 displays a size slightly smaller than F2, while F3 exhibits the smallest droplet size emphasizing the best emulsifying behavior. Incorporation of dimenhydrinate resulted in a reduction in size of the area of emulsion originating from formation at lower lipid/surfactant ratios (light gray area).

#### Stability studies of dimenhydrinate loaded formulations

As SEDDS are supposed to get emulsified in contact with the body fluids, a dilution of 1:2 could be assumed as realistic if the formulations are applied via the nose with only small quantity of liquid present on the mucosal surface. Consequently, pre-concentrates were diluted in Ringer’s solution in a volume ratio 1:1. Recorded parameters of the stability assessment over a 2-week storage time are listed in Table 2 and corresponding intensity distribution plots are shown in the supporting information (Fig. S6-S8). Polydispersity indices of the formulations were within an acceptable range, except for F3 where larger PDI indicates a higher heterogeneous dispersion. Significant increase in droplet size of F3 within the first 24 h was monitored (***P < 0.001). However, the formulation’s homogeneity was preserved afterwards up to 2 weeks. SEDDS formulations maintained moreover their integrity undergoing different temperature cycles. Neither phase separation, nor creaming or precipitation of dimenhydrinate was observed. Particle size, PDI, and the amount of drug load did not change significantly. Owing to these findings, SEDDS could be designated as physically stable under the applied storage conditions.

**Table 2:** Droplet size and polydispersity index of dimenhydrinate loaded SEDDS in a dilution of 1:2 in Ringer’s solution over 2 weeks of storage at 25 °C. Measurements were performed at 37 °C. Values are means with standard deviation (*n* = 3)

	Time	Particle size [nm]	PDI
F1	0 h	169.6 ± 1.9	0.24 ± 0.01
	3 h	172.5 ± 2.7	0.25 ± 0.01
	24 h	183.7 ± 9.7	0.29 ± 0.01
	2 weeks	183.4 ± 5.1	0.29 ± 0.03
F2	0 h	221.1 ± 0.6	0.26 ± 0.01
	3 h	228.6 ± 3.5	0.27 ± 0.01
	24 h	225.8 ± 3.6	0.27 ± 0.01
	2 weeks	227.3 ± 4.8	0.28 ± 0.03
F3	0 h	58.5 ± 1.6	0.30 ± 0.04
	3 h	66.4 ± 2.9	0.40 ± 0.02
	24 h	69.9 ± 1.7	0.41 ± 0.04
	2 weeks	72.9 ± 1.4	0.38 ± 0.06

#### Transmission electron microscopy

**Fig. 2:**
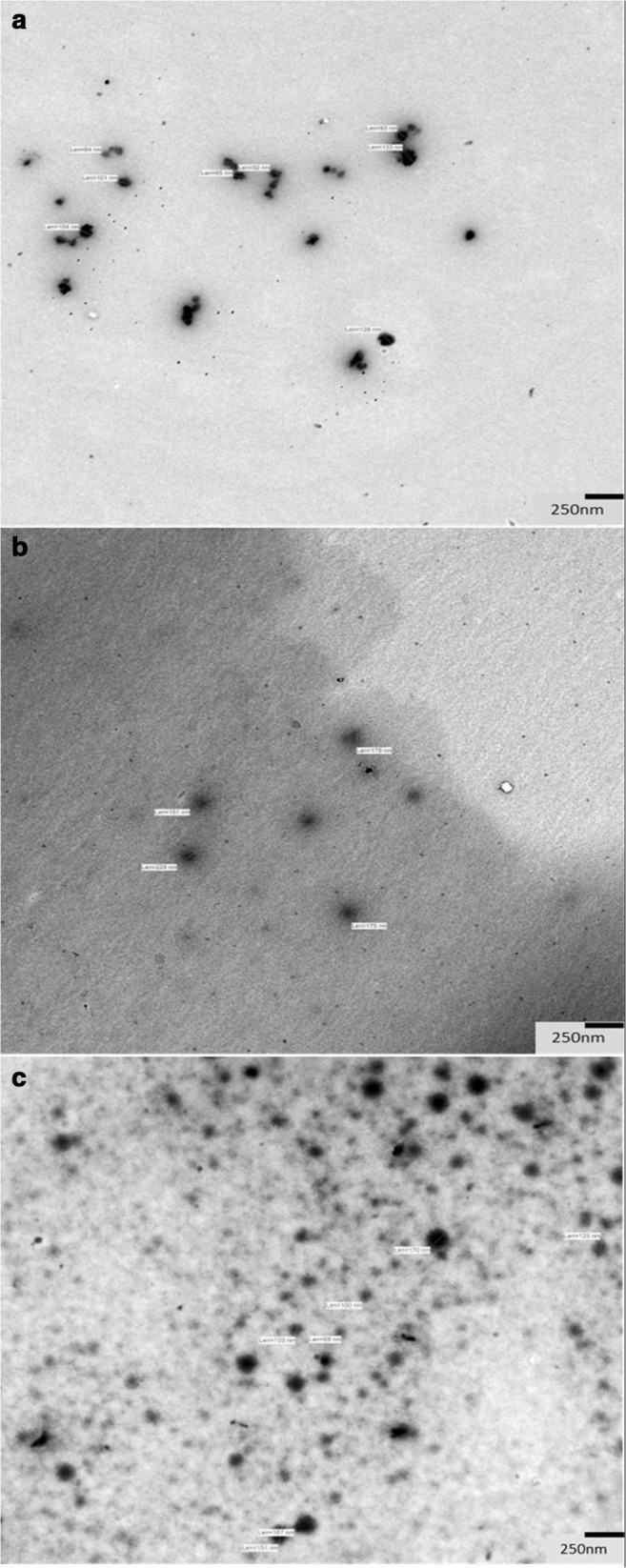
TEM images of F1 (**a**), F2 (**b**), and F3 (**c**)

Transmission electron microscopy (TEM) images are shown in Fig. 2 and the associated intensity distribution plots of the dynamic light scattering measurements are included in the supporting information (Fig. S9-S11). A broad size distribution of all emulsions is clearly visible (F1, ~ 60–140 nm; F2, ~ 80–230 nm; F3, ~ 50–180 nm). Nevertheless, a direct comparison with the data obtained by dynamic light scattering measurements is rather difficult, since the investigation of hydrated samples by conventional electron microscopy might cause structural alterations within the system induced by vacuum and the beam as well as by the drying process. Complete dehydration during sample preparation might result in effects like severe shrinkage, collapse, selective dimensional modification, and aggregation.

### Assessment of spreadability and sprayability

**Fig. 3:**
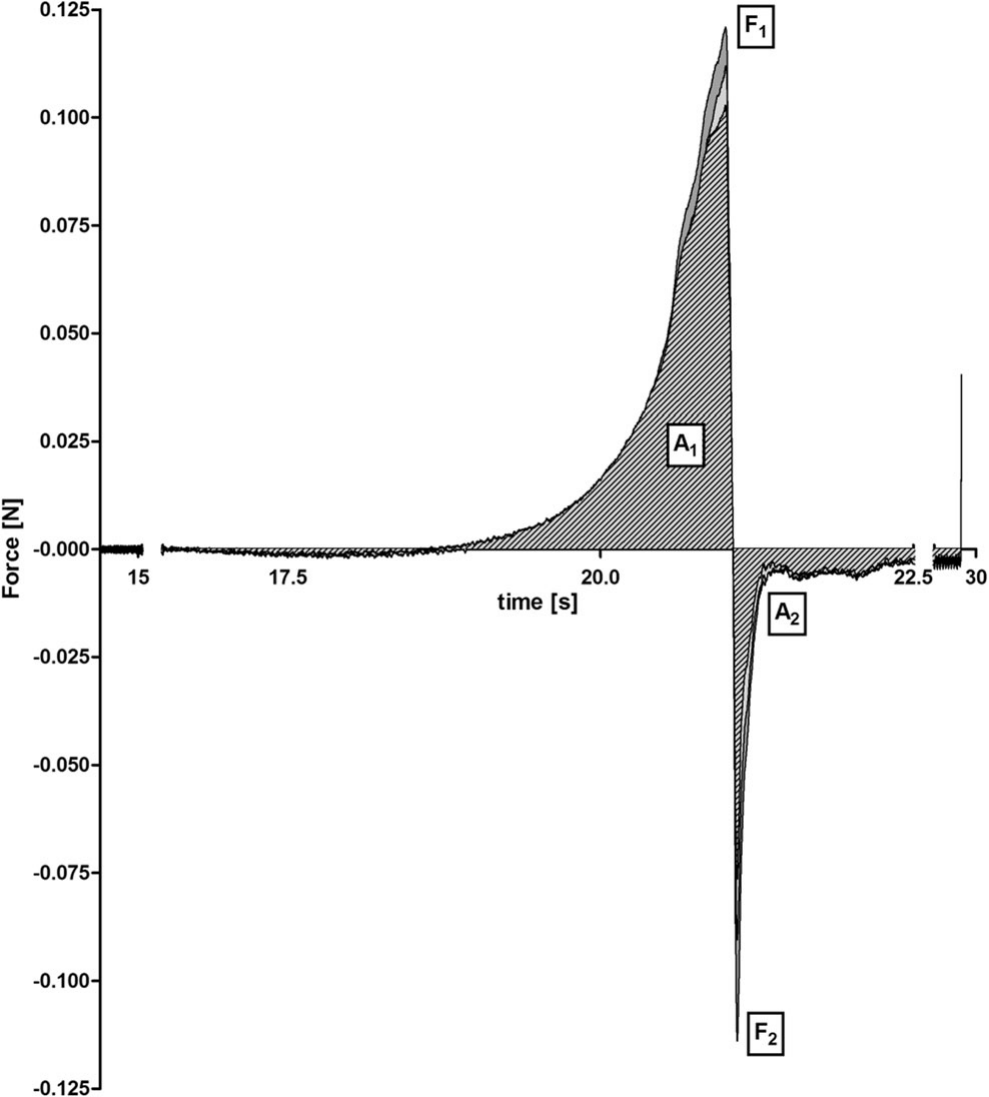
Force-time diagrams of the spreadability investigations of the pre-concentrates. The areas between the graphs and x-axis are filled as follows: light gray (F1), dark gray (F2), and light gray with striped pattern (F3). A_1_: work of spreading, F_1_: firmness, A_2_: work of adhesion, F_2_: stickiness

**Fig. 4:**
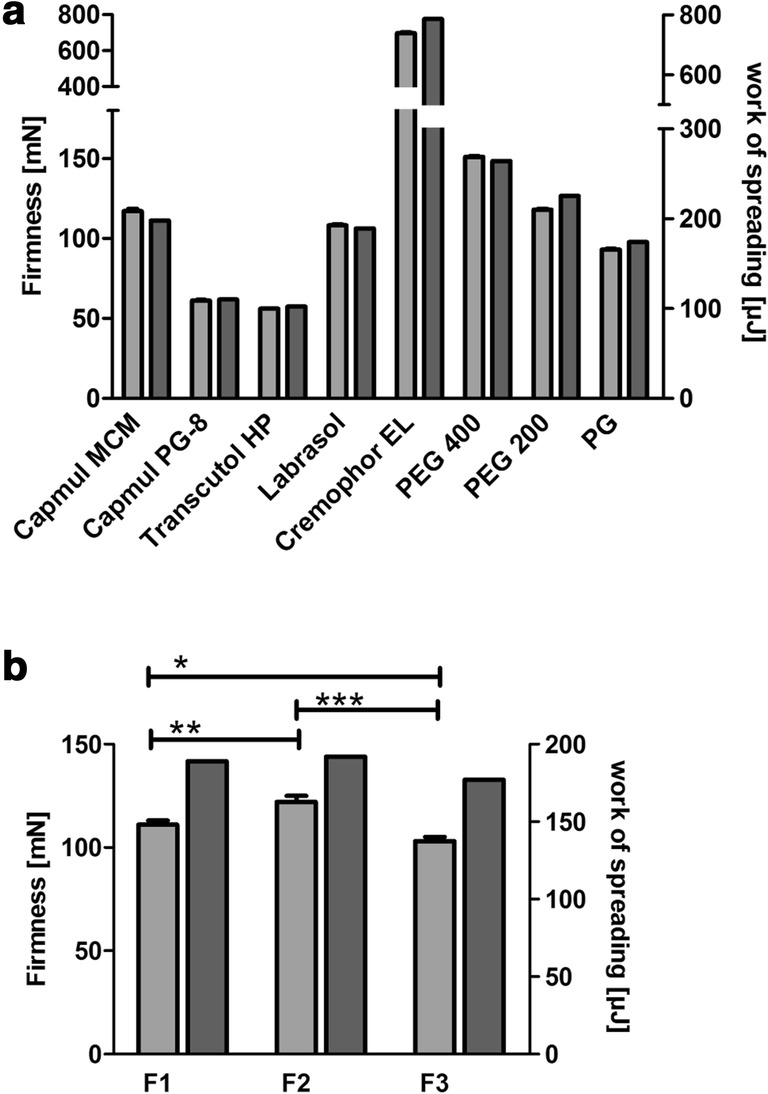
Assessment of the spreadability. Values are means of at least three experiments ± SD (*P < 0.05, **P < 0.01, ***P < 0.001). Light gray columns display the firmness and the work of spreading is represented by the dark gray columns. **a** Data of the single components. **b** Values of the SEDDS pre-concentrates of the formulations F1, F2, and F3

Since the spreading of a formulation is an important textural feature that governs the performance during application, as well as ensures uniform coverage of the mucosal surface, the extent of spreadability was explored. Force-time diagrams of the pre-concentrates are illustrated in Fig. 3. The maximum positive force (F_1_) represents the firmness of the sample and is measured at the maximum penetration reached at the final gap width of 2 mm. The work of spreading is defined as the total amount of force required to spread the formulation. Within the diagram, it is depicted by the area above the x-axis (A_1_). Basically, the lower the material’s firmness, the lower the work of spreading, and as a consequence, the substance possesses a thinner consistency. For reasons of comparability, similar results of the single pre-concentrates would be beneficial to guarantee an equal distribution at the application site. Having a look at the data obtained by the pre-concentrates, there is no difference in the work of spreading but firmness was significantly different (Fig. 4, part b). Characteristics of the SEDDS pre-concentrates are approximately in the range of those obtained by Labrasol and PG (Fig. 4, part a). Both excipients are reported to own excellent spreading properties [28]. By the fact that less firmness is related to a lower force needed to spread the formulation, a connection to better emulsification properties and smaller droplet size should be given [29]. Results of the size measurements are in agreement with this assumption as SEDDS size decreased in the order of F2, F1, and F3. Moreover, low firmness is facilitating the release of formulation from a dosing system leading to an improved sprayability. Findings of a sprayability experiment using a nasal spray confirmed an almost entire discharge of pre-concentrate per dose with 90.5 ± 1.8% for F1, 92.5 ± 1.9% in case of F2, and 100.3 ± 2.0% for F3. While the amount of recovered dimenhydrinate was higher in F3 than in the other concentrates (**P < 0.01), detection of any discrepancy between F1 and F2 was not possible.

### Drug release

Recently published literature discussed that the phenomenon of release can be best explained by the assumption of a simple diffusion process from the lipophilic phase (SEDDS droplets) into the aqueous phase (release medium). Consequently, drug release is mainly controlled by the partitioning coefficient (log D) of the drug between SEDDS and the release medium. Log D values of dimenhydrinate are represented in Table 3. Hence, the dimenhydrinate concentration remaining in SEDDS upon emulsification was calculated using eq. 3 in order to describe the formulations´ release behavior [30].

3 $$ C\ \mathrm{SEDDS}\ \left(\%\right)=\frac{100\%}{1+\kern0.5em \frac{\mathrm{VRM}}{\mathrm{VSEDDS}\ast \kern0.5em D\kern0.5em }} $$

**Table 3:** Partition coefficient (log D _SEDDS/release medium_) of dimenhydrinate between the lipophilic phase (SEDDS) and indicated release media

	Release medium	Log D _SEDDS/release medium_
F1	Water	1.66
	Ringer’s solution	1.57
	PBS	1.58
F2	Water	1.61
	Ringer’s solution	1.52
	PBS	1.54
F3	Water	1.61
	Ringer’s solution	1.52
	PBS	1.54

With the volume of SEDDS pre-concentrate and of the release medium represented by V_SEDDS_ and V_RM_. C_SEDDS_ stands for the drug concentration remaining in SEDDS and the solubility ratio is given by D.

The lower the log D value, the higher the percentage of drug immediately released from the droplets. Apart from the log D value, the volume ratio of SEDDS to release medium has a big impact on drug release. Assuming a dilution factor of 1:2 in consideration of the low amount of liquid available in the nasal cavity, in vivo calculated initial release fraction of the dimenhydrinate payload would correspond to 2.6% for F1 and 2.9% in case of F2 and F3. Hence, the delivery system should maintain its function in view of transporting the active ingredient and enhancing permeation. When the initially amount of released drug is getting absorbed from the mucosal membrane, a concentration gradient is generated and further drug can diffuse out of the oily droplets until equilibrium is reached again. Drug release from SEDDS is therefore primarily controlled by the absorption membrane and can be characterized by the permeability coefficient (P_app_) of the drug.

#### Permeation across bovine nasal mucosa

**Fig. 5:**
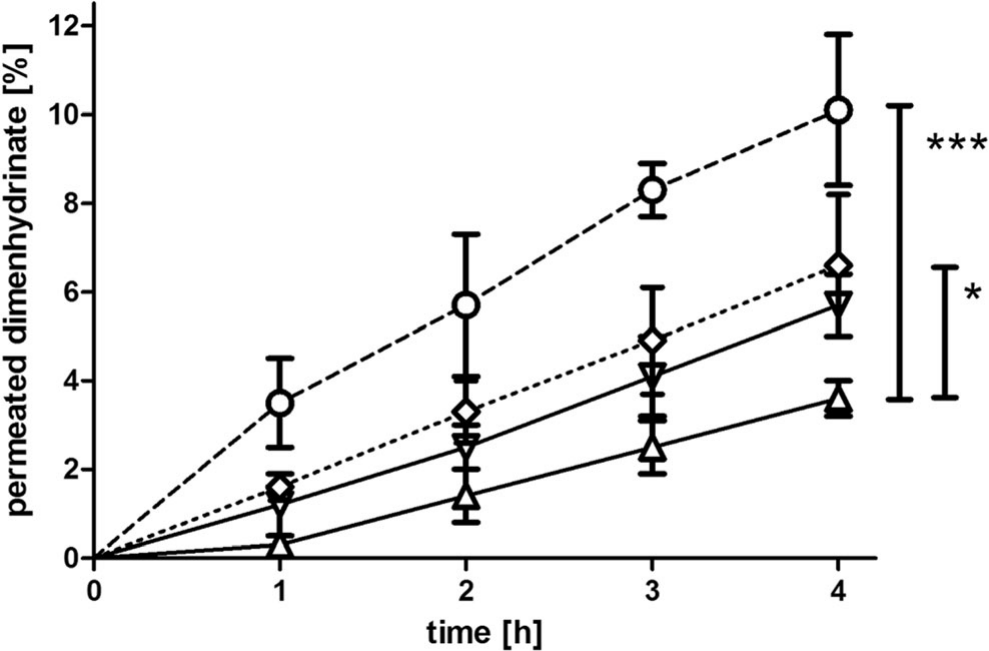
Mucosal permeation study of SEDDS. Graphs display the percentage of permeated dimenhydrinate across bovine nasal mucosa within 4 h incubation at 37 °C. Values represent data of at least three replications plus standard deviation (*P < 0.05, ***P < 0.001). F1 (**○**, dashed line), F2 (▽,solid line), F3 (◇, dotted line) and standard (△, solid line)

Studies on excised tissue from animals are regarded to mimic the in vivo situation to a highest degree. Bovine nasal mucosa is most frequently used, as it is easily available in high quantity and reproducible quality from local abattoirs and well suited for permeability studies [16]. Drug transport across the nasal mucosa was significantly improved upon incorporation in SEDDS compared to the control in Ringer’s solution by 2.8-fold (F1), 1.6-fold (F2), and 1.8-fold (F3), respectively (Fig. 5). Calculated permeability coefficients are listed in Table 4. Lin et al. investigated the nasal permeation of a series of anti-allergic drugs with different lipophilicity. Log *P* values of the tested compounds were within a range from − 1.58 to 3.21 with corresponding P_app_ values between 0.20 × 10^−6^ cm/s and 21.92 × 10^−6^ cm/s [31]. The experimentally determined log P of dimenhydrinate was 0.63, and the permeability coefficient was in good agreement with the outlined data of the mentioned study. Emphasizing log P as one of the most important factors affecting permeability, increase of lipophilicity of the drug vehicle seems to contribute to improved permeation. In addition, Transcutol HP, Labrasol, and Cremophor EL are known to enhance permeation [32, 33].

**Table 4:** The apparent permeability values (*P*_*app*_ values [cm/s]) of dimenhydrinate applied in the SEDDS formulations and in Ringer’s solution (standard). Depicted values represent means of three replications plus standard deviation

	*P*_*app*_ [cm/s]	
	Mean	SD
F1	10.91 × 10^−6^	1.80 × 10^−6^
F2	6.22 × 10^−6^	0.74 × 10^−6^
F3	7.16 × 10^−6^	1.76 × 10^−6^
Standard	3.91 × 10^−6^	0.33 × 10^−6^

### Evaluation of tissue toxicity

**Fig. 6:**
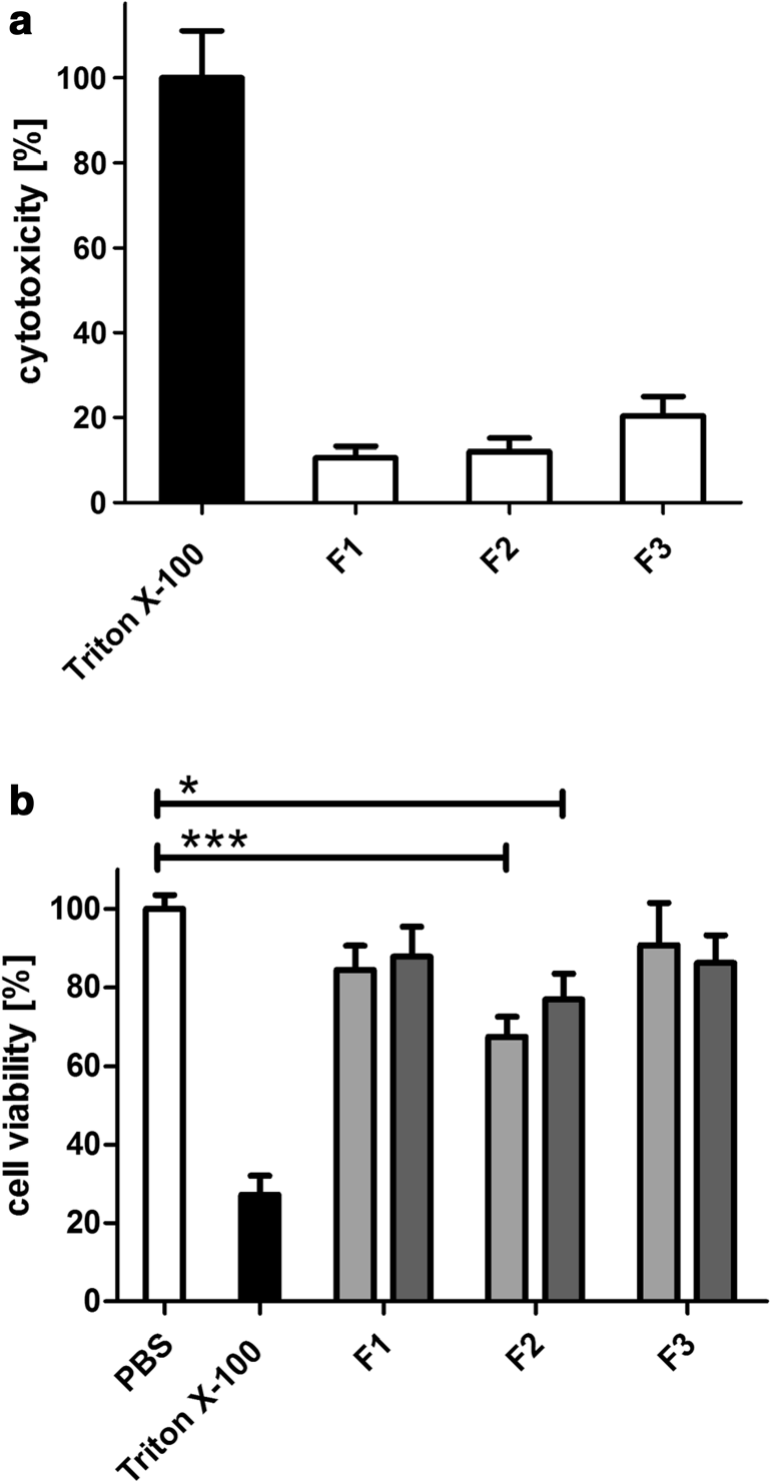
Evaluation of the toxicity of SEDDS to bovine nasal tissue. **a** Percentage of cytotoxicity determined performing LDH assay after the permeation study. **b** Assessment of cell viability with resazurin after 4 h incubation of bovine nasal mucosa with SEDDS in dilutions 1:2 (light gray) and 1:50 (dark gray). Values are means of at least three experiments ± SD (*P < 0.05, **P < 0.001)

Viability testing is a crucial requirement for experiments with excised tissue in order to ensure transferability of the outcome to a possible in vivo behavior. Moreover, cytotoxicity-related damages by drugs and permeation enhancers can be detected. To unquestionably guarantee viability, considering a combination of tests based on different mechanisms is favorable. Hence, tissue viability was explored on the one hand by conducting LDH membrane integrity assay and on the other hand by the resazurin assay. According to literature, collected tissue can maintain its vital status over at least 3–4 h up until 8 h after slaughter [16, 34]. In case of the controls in pure Ringer’s solution, as well as in PBS, no tissue toxic effects could be observed. All included surfactants are known to possess permeation enhancing effects and might provoke cell membrane interference combined with reduced viability. Regarding the high concentration of surfactants used, viability of tissue treated with the formulations was still in an acceptable range above 80% after the permeation experiment, confirming the integrity of the tissue (Fig. 6, part a). Additionally, the constant permeability rate of the formulations could be accounted as a viability feature (Fig. 5). Results of the resazurin assay were in good agreement to this, solely viability of the tissue samples incubated with F2 was significantly reduced compared to the negative control (Fig. 6, part b). A concentration dependency of the effect could not be ascertained.

**Fig. 7:**
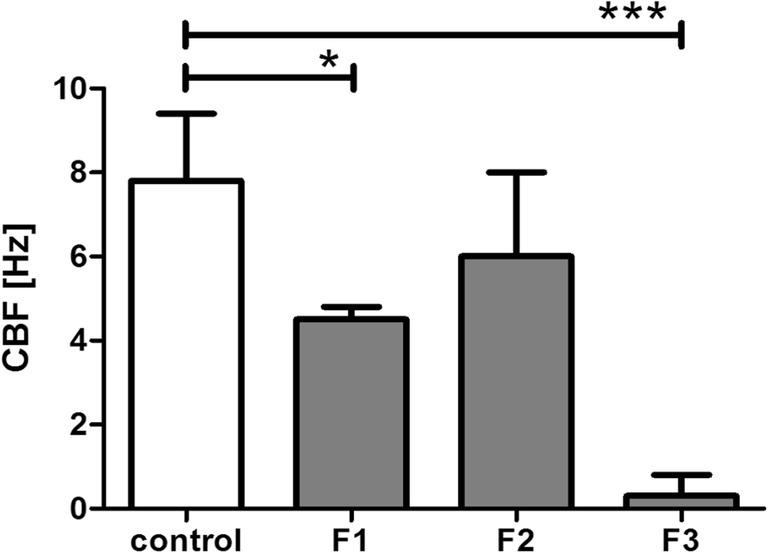
Ciliary beat frequency (CBF) of the control (initial beating frequency) and the recorded samples after treatment with the formulations and wash-off with Ringer’s solution. Values are means of at least three analyses ± SD (*P < 0.05, **P < 0.001)

Furthermore, nasal irritancy was investigated by CBF measurements. Determined basal CBF of the untreated control was 7.8 ± 1.6 Hz. Ciliary beating at 7–12 Hz can be assumed as the normal range after removal of ciliated cells by nasal brushing [35]. Furthermore, calculated values correlated well with frequencies of cultured human ciliated cells of a previous study being in the range of around 8–12 Hz [36]. CBF of the control, as well as of the ciliated cells after incubation with the formulation pre-concentrates, are shown in Fig. 7. According to the categorization of cilio-inhibiting effects introduced by Merkus et al. [37], formulations are considered as cilio-friendly if cilia exhibit at least 75% of their initial frequency after formulation wash-off, whereas cilio-inhibition is expected between 25 and 75%. Ciliostasis is defined at frequencies less than 25% of the basal CBF. Consequently, CBF of F2 was not statistically significant different to the control (87.7 ± 26.2%). F1 turned out to be cilio-inhibiting (58.2 ± 4.0) and application of F3 evoked complete ciliostasis (4.3 ± 6.0%). The more pronounced effect of F3 as compared to F1 and F2 might be explained by the higher amount of hydrophilic surfactants which could provoke impairments of the sensitive ciliary membrane. Nevertheless, effects on in vitro CBF are not fully transferable to in vivo situation by reason of different physiological parameters which cannot be simulated. First of all, cilia might be more protected by the continuously produced mucosal secretions (around 1.5–2 L nasal mucus per day) in combination with clearance from the administration site under an average velocity of 5 mm/min, resulting in a reduction of the amount of formulation being in direct contact with the ciliated cells [38]. Secondly, the nasal epithelium can be expected to recover from damage as epithelial cells are permanently replaced by fresh cells at the basement membrane [21]. Furthermore, formulations are intended for acute treatments rather than for chronic treatments.

## Conclusion

A couple of studies have already been performed for the intranasal administration of antiemetics with metoclopramide, granisetron, or ondansetron as the most frequently investigated candidates [39]. Applied strategies in order to improve drug delivery focused on either extending the residence time at the mucosal surface by using polymers, or on improving permeability via including permeation enhancers or encapsulation in particulate carriers. Efficacy of the mentioned concepts was verified by profound in vitro and in vivo studies. Within the present study, it was therefore the aim to create a self-emulsifying drug delivery system suitable for the nasal application of dimenhydrinate. Hence, well-known nasal permeation enhancers like Transcutol HP, Labrasol, or PEG 400 were included in the formulations to facilitate drug diffusion of the submicron-sized droplets across the mucosal barrier on the one hand, besides working as a solubilizer for dimenhydrinate on the other hand. Key findings of the undertaken experiments stated a high payload owing to the improved drug solubility and an enhanced ex vivo permeation compared to the control. Viability assays showed minor effects on account of the high amounts of surfactants. On the base of this data, it can be concluded that the exerted approach could be an important step in the investigation of SEDDS for the intranasal delivery of dimenhydrinate.

## Electronic supplementary material

ESM 1 (PDF 1663 kb)
