# Supplementary material for: In vitro evaluation of a self-emulsifying drug delivery system (SEDDS) for nasal administration of dimenhydrinate
Source: Drug Deliv Transl Res. 2019 Mar 14;9(5):945–55. doi: 10.1007/s13346-019-00634-1 (PMC6731199; doi:10.1007/s13346-019-00634-1)
Supplement: Supplementary file 1 — (PDF 1663 kb) [file 13346_2019_634_MOESM1_ESM.pdf]

Supplementary Material

In vitro evaluation of a self-emulsifying drug delivery system (SEDDS) for nasal administration of dimenhydrinate

Drug Delivery and Translational Research

Christina Leichner[1], Randi Angela Baus[1], Max Jelkmann[1], Melanie Plautz[1], Jan Barthelmes[1], Sarah Dünnhaupt[1] and Andreas Bernkop-Schnürch[1 *]

[1]Center for Chemistry and Biomedicine, Department of Pharmaceutical Technology, Institute of Pharmacy, University of Innsbruck, Innrain 80/82, 6020 Innsbruck, Austria

***Corresponding Author**: andreas.bernkop@uibk.ac.at

Tel.: +43-512-507 58601 Fax: +43-512-507 58699

HPLC method development for dimenhydrinate

The calibration curves of dimenhydrinate at wavelength of 225 nm and 273 nm are shown in Figure S1 and S2. Associated values are listed in Table S1 and S2.

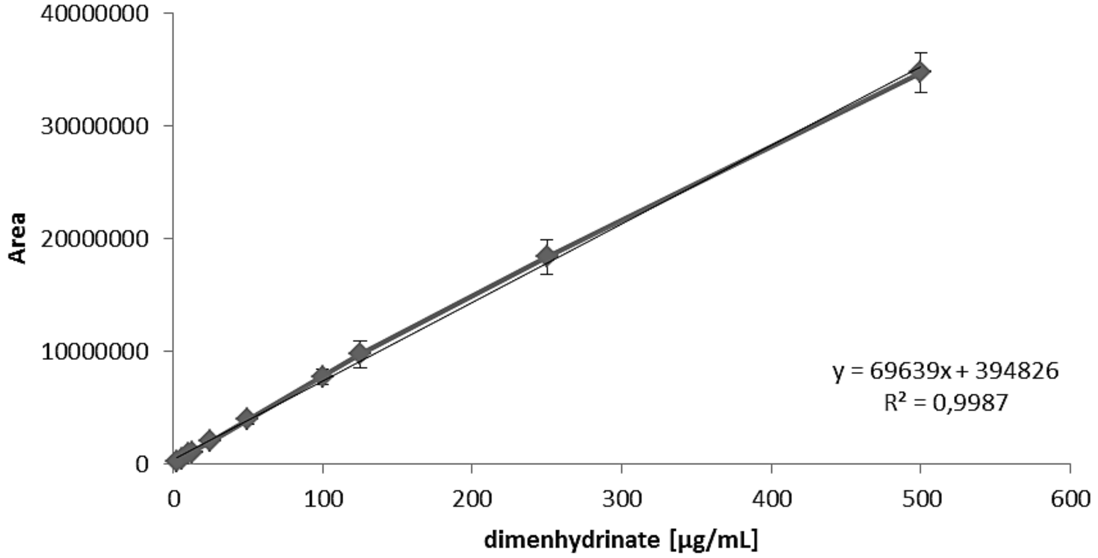

Fig. S1 Calibration curve of dimenhydrinate at a wavelength of 225 nm. Data points represent the mean values of three replications plus standard deviation.

Table S1 Concentration of dimenhydrinate and the corresponding peak area at a wavelength of 225 nm. Data are represented by the mean values of three replications plus standard deviation.

	**Dimenhydrinate [μg/mL]**	**Area**	
		**Mean**	**SD**
**A**	500	34716824	1746103
**B**	250	18348269	1487836
**C**	125	9723797	1145147
**D**	100	7759122	692848
**E**	50	3970611	383810
**F**	25	2035320	203091
**G**	12.5	1049391	145799
**H**	6.25	460480	48413
**I**	3.125	237950	22552

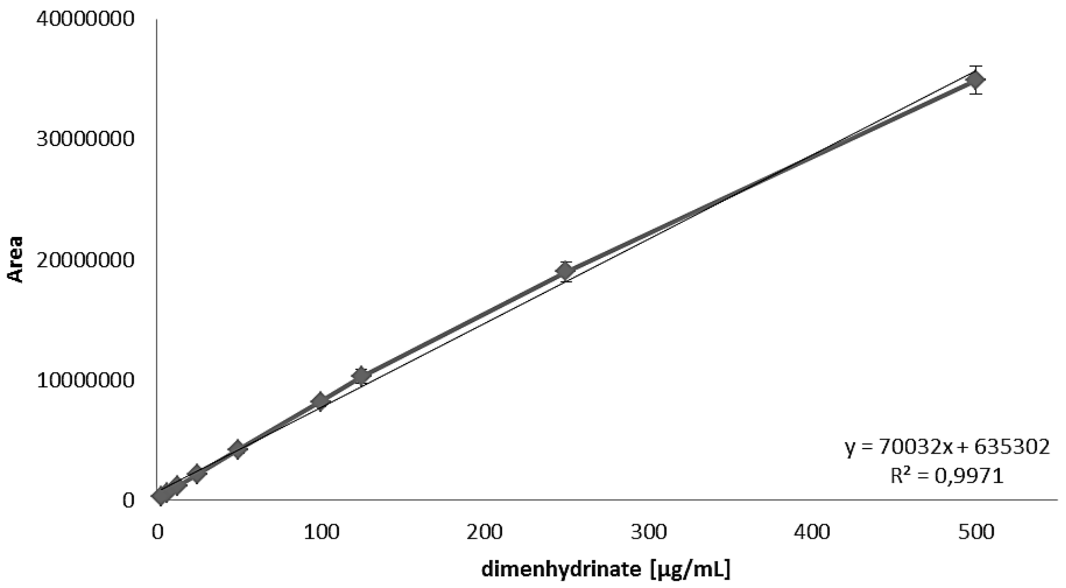

Fig. S2 Calibration curve of dimenhydrinate at a wavelength of 273 nm. Data points represent the mean values of three replications plus standard deviation.

Table S2 Concentration of dimenhydrinate and the corresponding peak area at a wavelength of 273 nm. Data are represented by the mean values of three replications plus standard deviation.

	Dimenhydrinate [µg/mL]	Area	
		Mean	SD
**A**	500	34916904	1158995
**B**	250	18994853	841489
**C**	125	10293077	599689
**D**	100	8168998	352232
**E**	50	4197174	256615
**F**	25	2175845	100638
**G**	12.5	1136811	66625
**H**	6.25	589143	28419
**I**	3.125	310688	21138

Solubility of dimenhydrinate

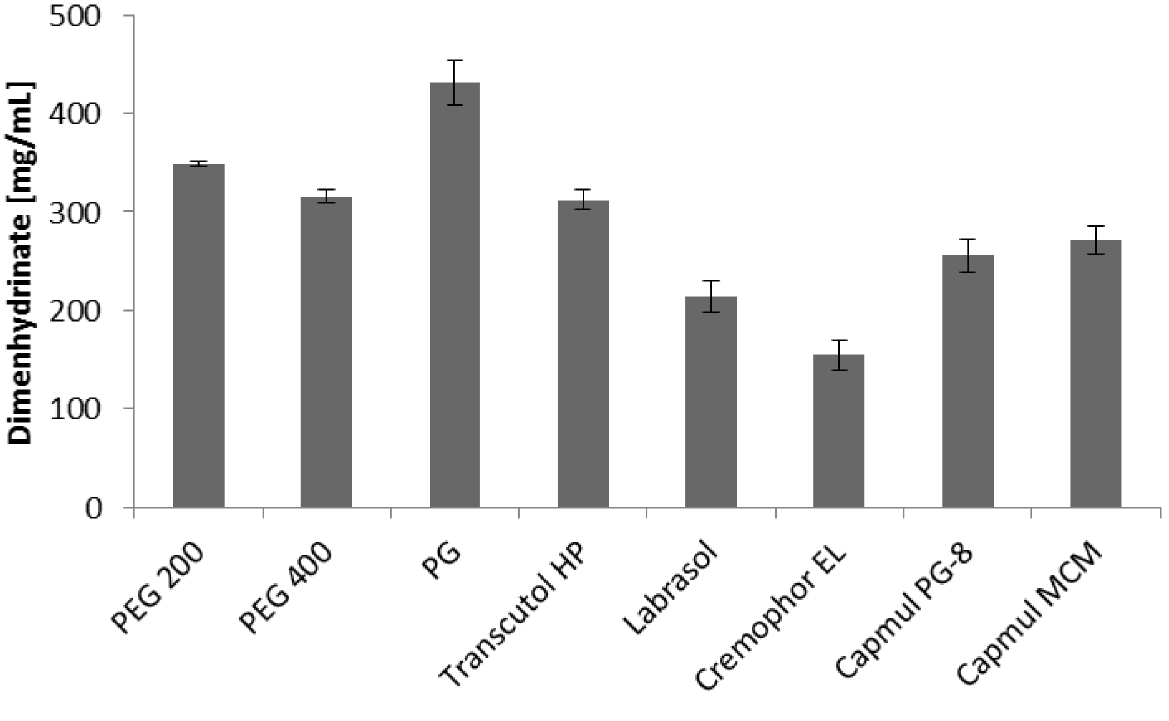

Fig. S3 Solubility of dimenhydrinate in the single components of the SEDDS pre-concentrates. Values are means of three measurements plus standard deviation.

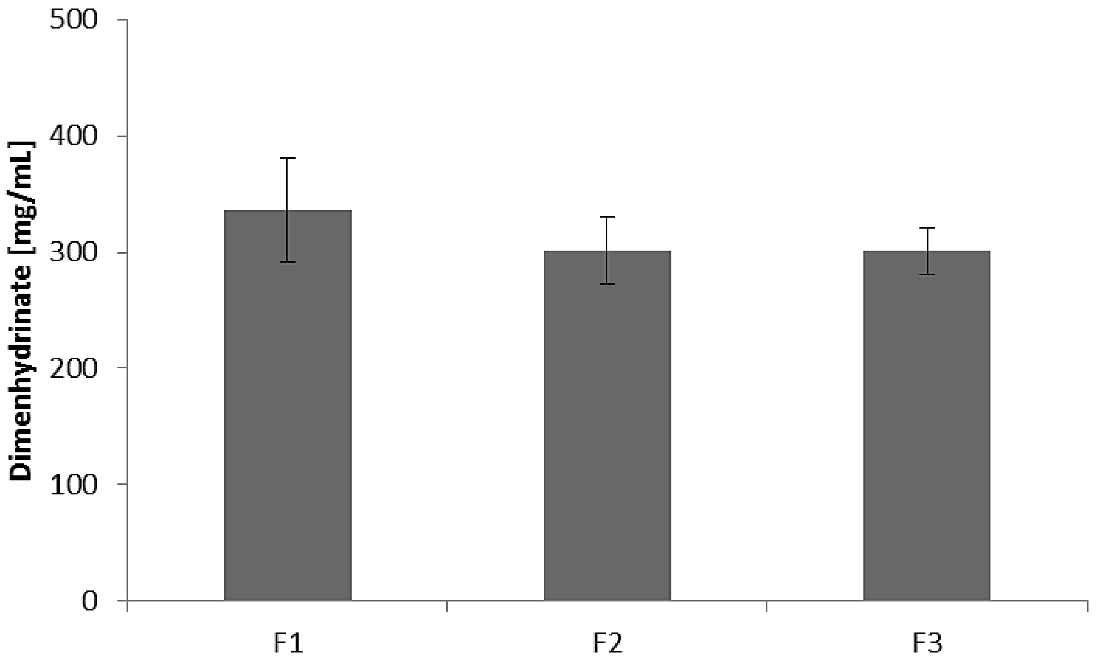

Fig. S4 Maximum solubility of dimenhydrinate in the SEDDS pre-concentrates. Values are means of three measurements plus standard deviation.

Composition of SEDDS pre-concentrates- preliminary investigations

Selected excipients were combined in different volume ratios and investigated visually for stability. A variety of pre-concentrate compositions from preliminary tests is shown in Table S3 as well as the resulting particle size and visual appearance of 1:2 dilutions in Ringer´s solution. Dimenhydrinate possessed the highest solubility in Transcutol HP (HLB 4) followed by Capmul MCM (HLB 5.5) and Capmul PG-8 (HLB 6.7). Accordingly, these three components were included into the formulations to build the oily vehicle for the drug. Compositions with oily phase in a range of 40-60 % with Cremophor EL as hydrophilic surfactant and PEG 200 or PEG 400 as co-surfactant were not stable. Stability could be improved by combining Labrasol and Cremophor EL. Exclusion of co-surfactants resulted in good emulsifying properties using a combination of 30 % Cremophor EL and Labrasol for an amount of 40 % oily phase. Nevertheless, because of fluctuations in droplet size over 24 h

storage of the formed emulsions, the addition of co-surfactants was decided in order to support the formation of emulsion droplets and preserve stability of the emulsions.

Table S3 Compositions of investigated pre-concentrates including size characteristics and appearance of 1:2 emulsions in Ringer´s solution.

	Components [%]									
	Transcutol HP	Capmul MCM	Capmul PG-8	Labrasol	Cremophor EL	PEG 200	PEG 400	PG	Particle size	Appearance
**1**	20	40			20		20		> 6000 nm	milky
**2**	25	30			30		15		720 nm; PDI 0.8	milky
**3**	20	35			30		15		1107 nm; PDI 1	milky
**4**	30		20		40		10		~ 500 nm PDI 0.9	milky
**5**	20	20			40		20		~ 500 nm PDI 0.8	milky
**6**	25		25		30	20			200 nm PDI 0.7	turbid, bluish
**7**	20		20	20	20	20			600 nm PDI 0.6	milky
**8**	20		25	20	25	10			536 nm PDI 0.6	milky
**9**	20	25		15	30	10			300 nm PDI 0.4	turbid, bluish
**10**	15		25	35	25				180 nm PDI 0.2	turbid, bluish
**11**	25		25	20	30				130 nm PDI 0.3	turbid, bluish
**12**	25	25		20	30				122 nm PDI 0.3	turbid, bluish
**13**	20	25		25	30				100 nm PDI 0.3	turbid, bluish
**14**	20		25	25	30				100 nm PDI 0.3	turbid, bluish
**15**	20	25		30	25				65 nm PDI 0,4	bluish
**16**	15	25		30	30				55 nm PDI 0,4	bluish
**17**	20		20	30	30				67 nm PDI 0.3	bluish
**18**	20	20		30	30				67 nm PDI 0.4	bluish
**19**	20	10		30	20		20		100 nm PDI 0.2	turbid, bluish
**F1**	15	20			25	20		20	170 nm PDI 0.2	turbid, bluish
**F2**	15		15		30		20	20	220 nm PDI 0.3	turbid, bluish
**F3**	20	10		30	20		10	10	60 nm PDI 0.3	bluish

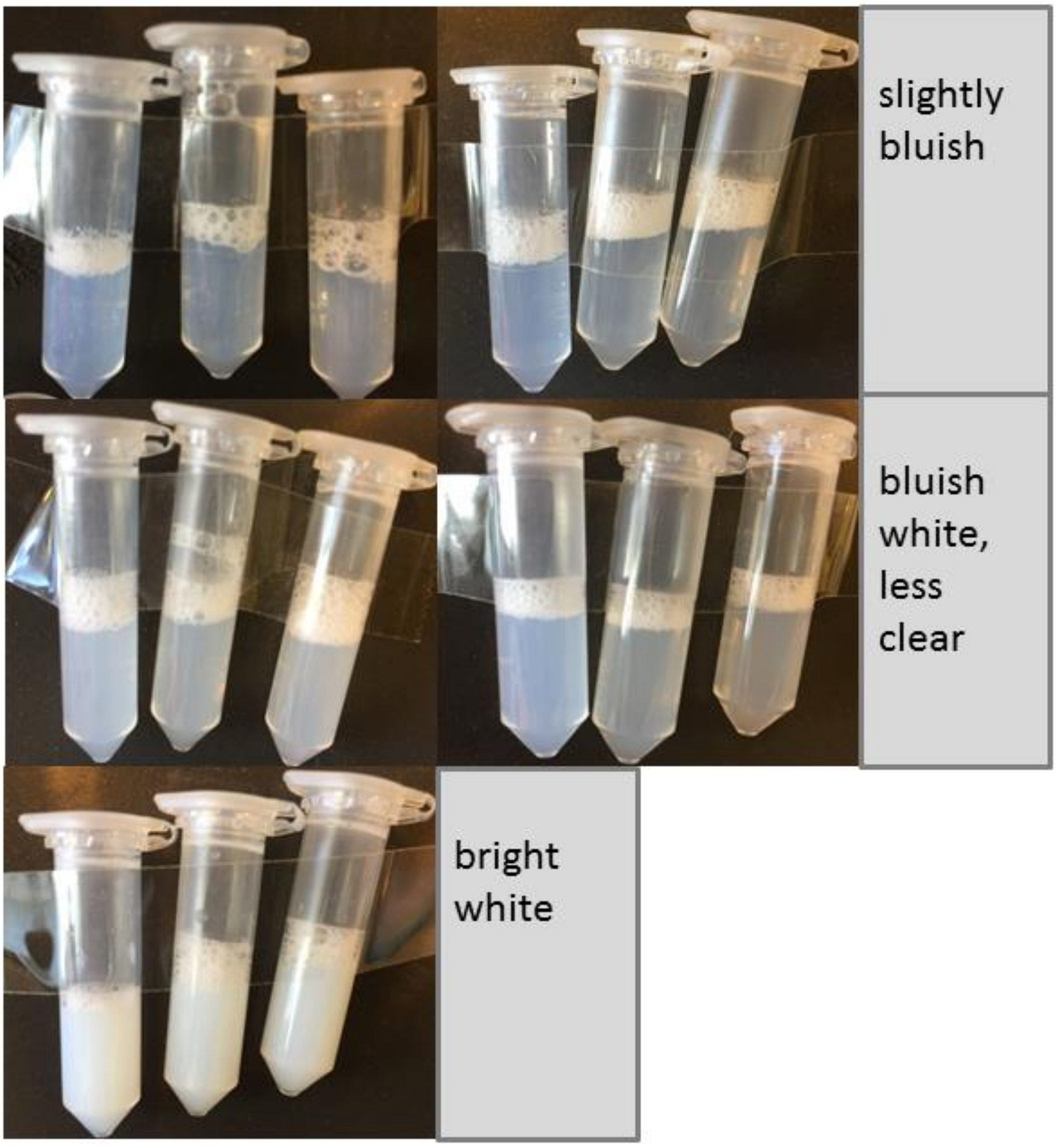

Fig. S5 Photographs illustrating exemplarily the visual appearance of mixtures classified as emulsions.

Droplet size measurements

Intensity distribution plots of the dynamic light scattering measurements of all emulsions are illustrated in Figure S6-S8. Size was recorded over a period of two weeks at a storage temperature of 25 °C. Outlined data belong to the mean values listed in Table 2 of the main article.

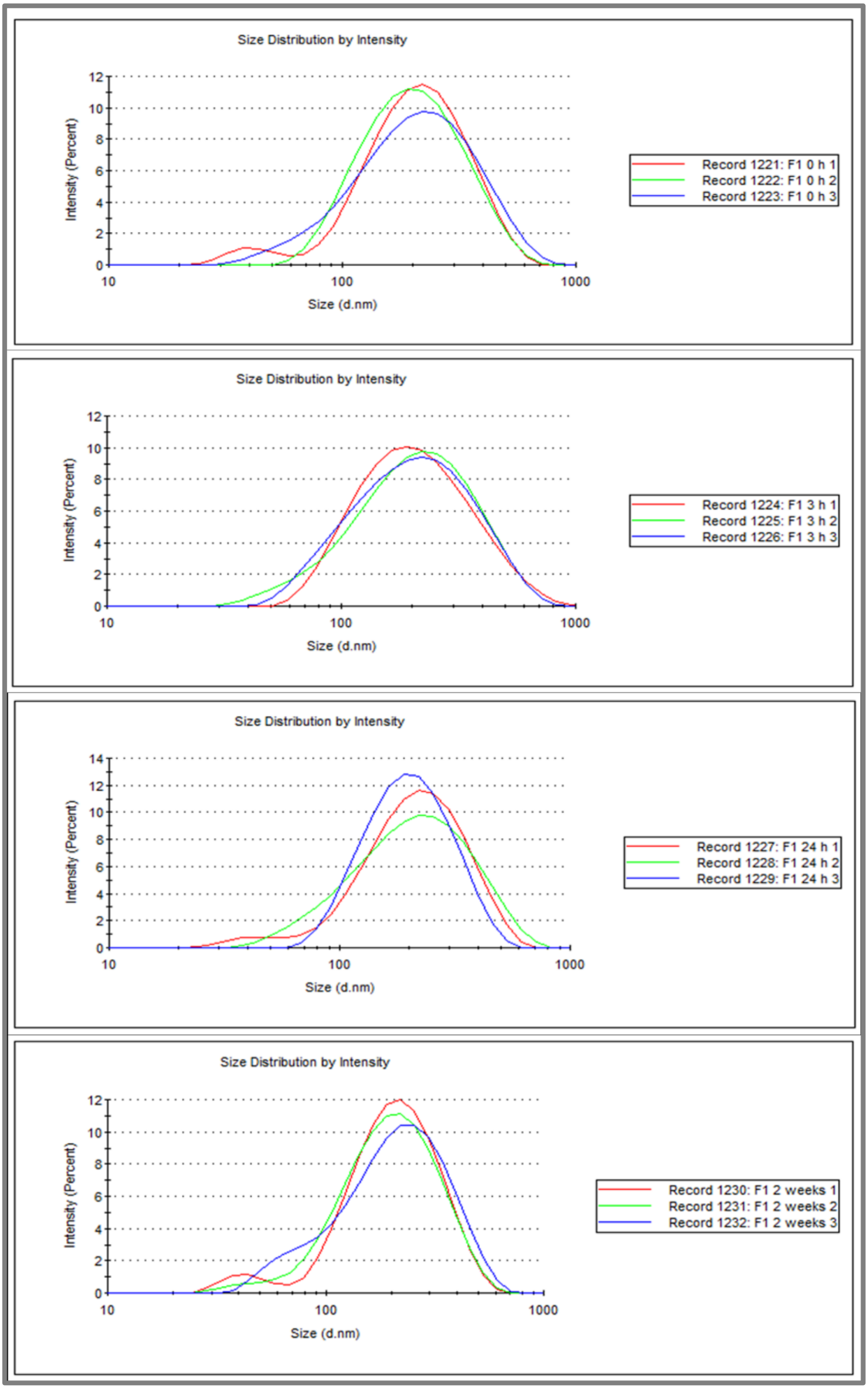

Fig. S6 Intensity distribution of particle size of SEDDS F1 diluted 1:2 in Ringer´s solution.

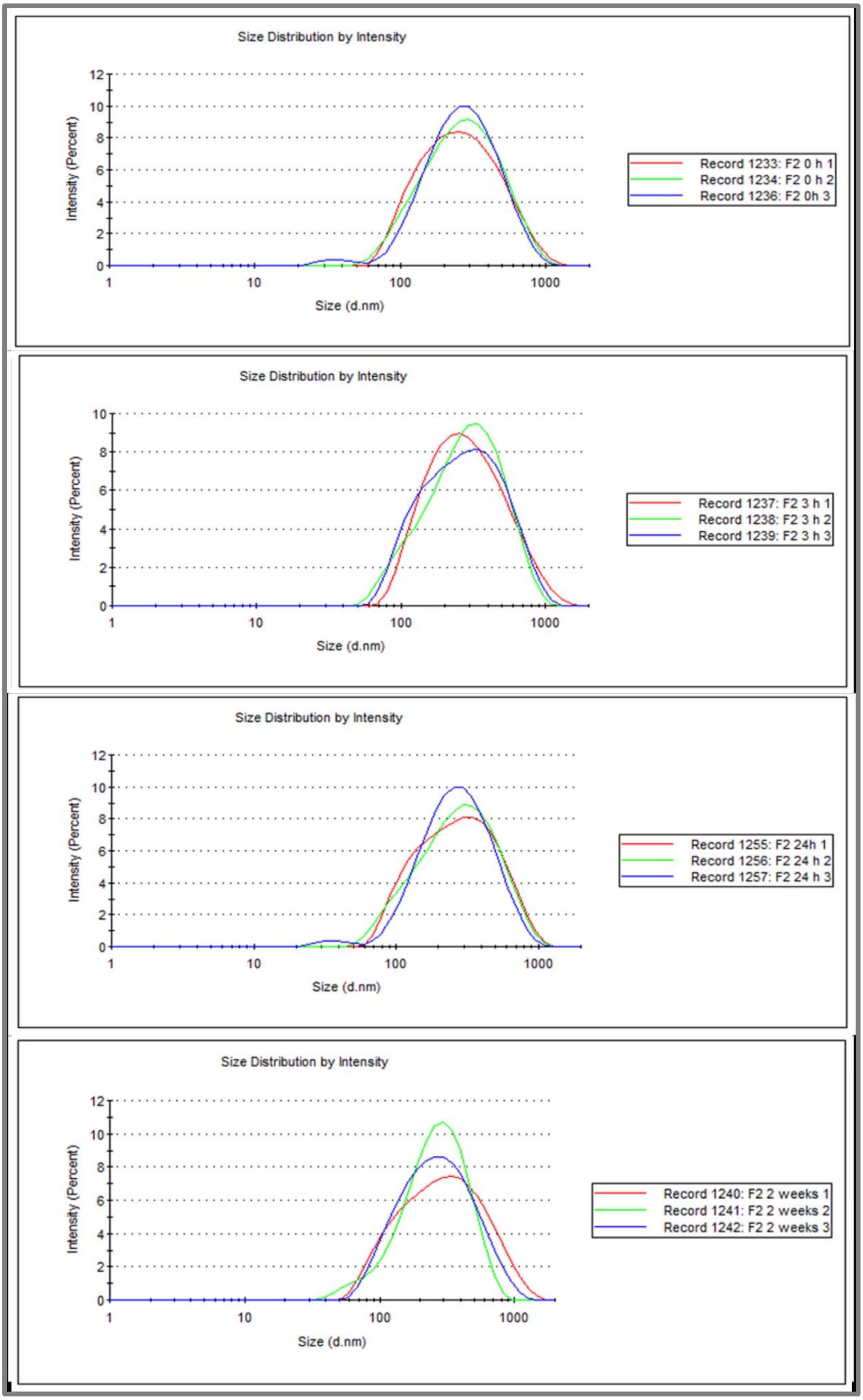

Fig. S7 Intensity distribution of particle size of SEDDS F2 diluted 1:2 in Ringer´s solution.

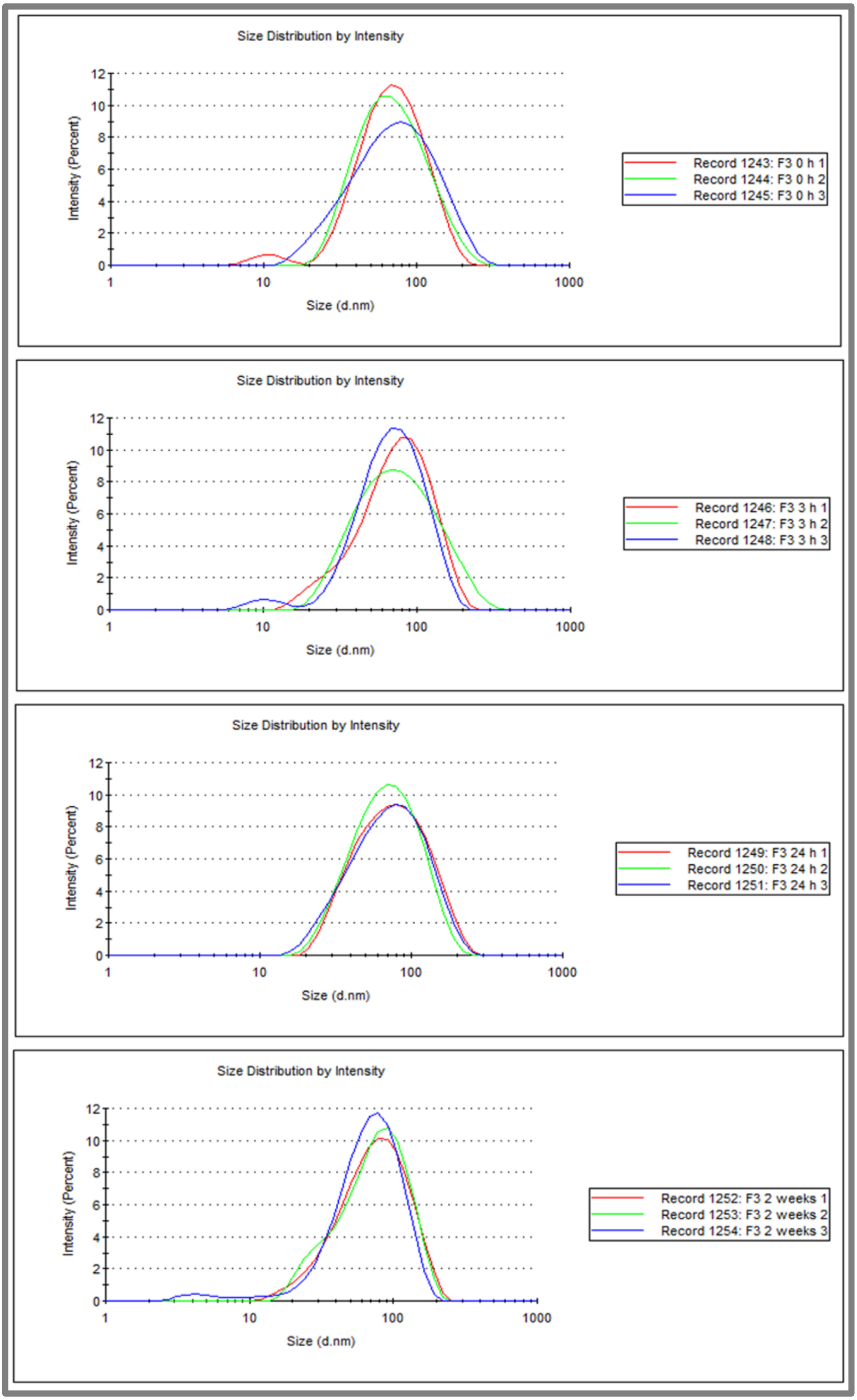

Fig. S8 Intensity distribution of particle size of SEDDS F3 diluted 1:2 in Ringer´s solution.

Intensity distribution plots of the dynamic light scattering measurements of 1:100 dilutions in demineralized water (Figure S9-S11).

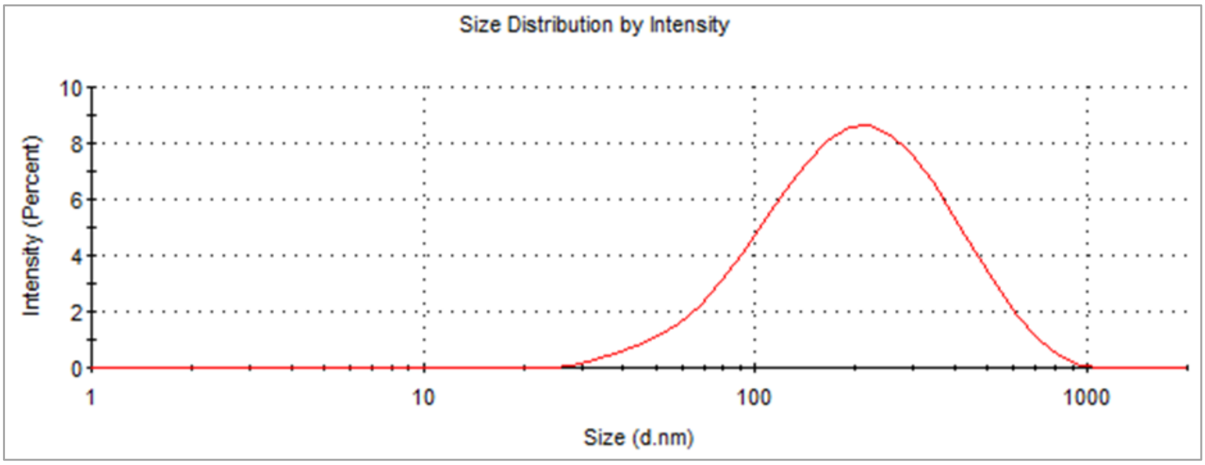

Fig. S9 Intensity distribution of particle size of SEDDS F1 diluted 1:100 in demineralized water.

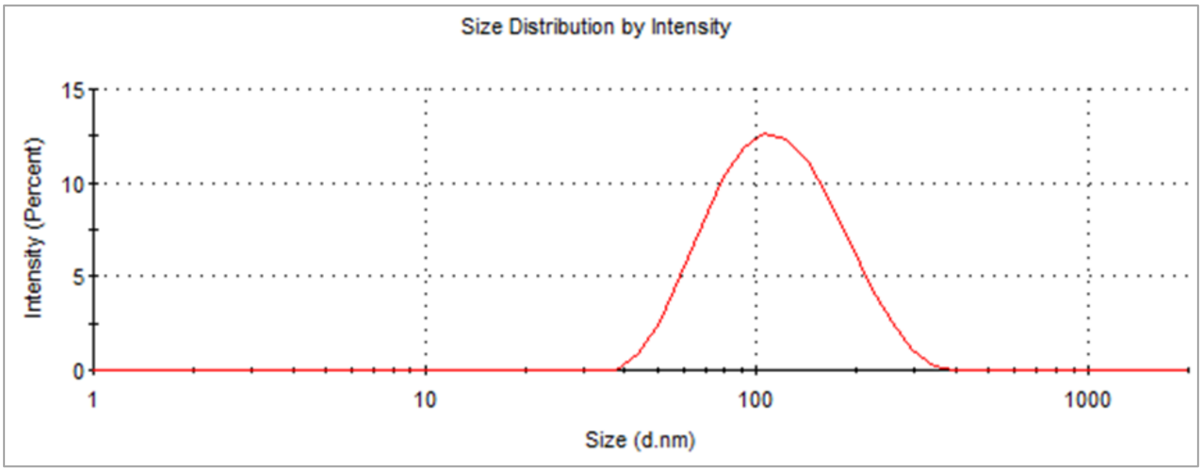

Fig. S10 Intensity distribution of particle size of SEDDS F2 diluted 1:100 in demineralized water.

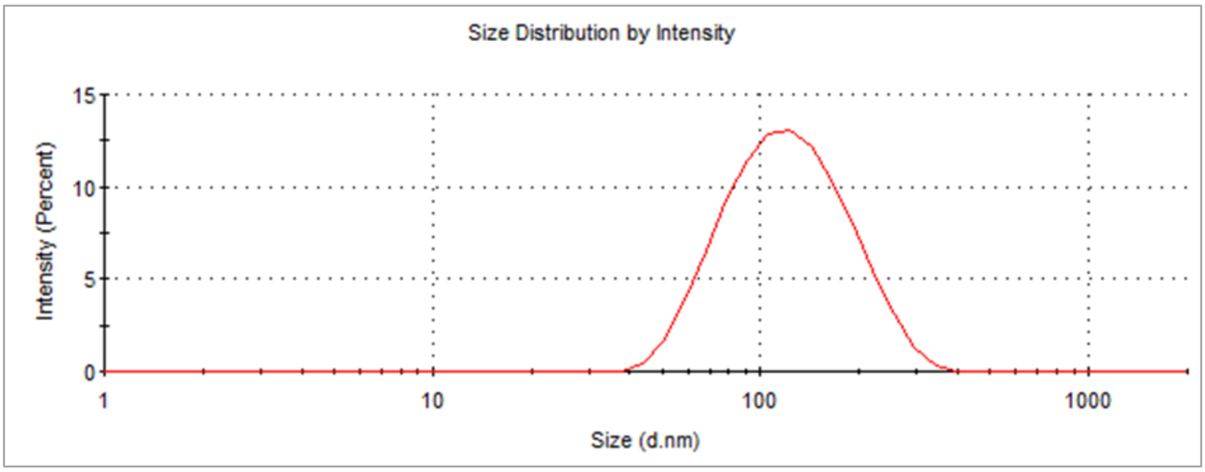

Fig. S11 Intensity distribution of particle size of SEDDS F3 diluted 1:100 in demineralized water.
